# Development and Validation of a Tool to Explore Attitudes Towards meDication adHErence Using a Novel Self-Reported QuestionnairE (ADHERE-7)

**DOI:** 10.3390/pharmacy12040113

**Published:** 2024-07-18

**Authors:** Iva Bužančić, Mislav Balen, Dahna Arbanas, Slaven Falamić, Katarina Fehir Šola, Ana Galić Skoko, Mirna Momčilović, Ante Orbanić, Alena Tatarević, Maja Ortner Hadžiabdić

**Affiliations:** 1 City Pharmacies Zagreb, 10 000 Zagreb, Croatia; 2 Faculty of Pharmacy and Biochemistry, University of Zagreb, 10 000 Zagreb, Croatia; 3 Karlovac Pharmacy, 47 000 Karlovac, Croatia; 4 Faculty of Medicine, Josip Juraj Strossmayer University, 31 000 Osijek, Croatia; 5 ZU Ljekarna Bjelovar, 43 000 Bjelovar, Croatia; 6 Zagreb County Pharmacies, 10 000 Zagreb, Croatia; 7 University Hospital Center, 10 000 Zagreb, Croatia; 8 Pharmacy Bobanović Vujnović, 52 100 Pula, Croatia

**Keywords:** medication adherence, polypharmacy, tool development and validation

## Abstract

Despite the availability of various tools for measuring medication adherence, efficiently identifying non-adherence levels and reasons at the point of care remains challenging. Existing tools often lack the ease of use needed for practical clinical application. This study aimed to develop and validate a user-friendly tool to provide healthcare professionals with a concise yet comprehensive means of identifying adherence behaviors. The methodology consisted of two phases: tool items were first developed using the nominal group technique with healthcare professionals, followed by a cross-sectional pilot study involving community-dwelling adults in Croatia. Validation analysis indicated acceptable face and content validity and satisfactory criterion validity, with Attitudes towards meDication adHErence self-Reported questionnairE (ADHERE-7) scores correlating with both the self-reported five-item Medication Adherence Report Scale (MARS-5 tool) (ρ = 0.765; *p* < 0.001) and an objective measure of the proportion of days covered (PDC) from pharmacy prescription claims data (G = 0.586; *p* = 0.015). Construct validity revealed three factors: Aversion, Comfort, and Practical Non-Adherence, with Cronbach’s alpha values of 0.617 for Aversion and 0.714 for Comfort Non-Adherence. The mean total score for ADHERE-7 was 26.27 ± 2.41 (range 17 to 28). This robust validation process confirms the ADHERE-7 tool as a reliable instrument for assessing medication adherence, addressing aversion, comfort, practical issues, and both intentional and unintentional nonadherence.

## 1. Introduction

Medication adherence can be defined as the degree to which the patient’s behavior regarding the use of medications corresponds with the agreed recommendations from a healthcare provider. Lack of and problems with medication adherence remains a longstanding problem patients and healthcare providers face daily [1,2]. 

Despite the availability of many effective drugs and evidence-based guidelines for treating chronic diseases, medication nonadherence affects 20–50% of patients on chronic medication, depending on the definitions used [3,4]. Inconsistent data are reported for Croatian patients’ levels of medication adherence. When explored using an anonymized self-reported tool, average adherence to long-term medication was around 42%, while examination of primary physicians’ prescribing records indicated around 70% of chronically ill patients were adherent [5,6]. Efforts are being made by the Croatian government and the national health insurance fund to introduce a new system for monitoring the outcomes of treatment, including adherence of outpatient chronic patients in community pharmacies, emphasizing the problem of non-adherence and the need for implementation of medication adherence-enhancing interventions [7]. It is estimated that nonadherence is associated with nearly 200,000 deaths and EUR 80–125 billion in potentially preventable direct costs (such as hospitalizations and medication waste) and indirect costs (such as lost work productivity) in the European Union (EU) alone [8]. Over time, various measures have been proposed to improve adherence [9], and initiatives and networks dedicated to adherence continue to recognize and prioritize the issue of medication nonadherence (i.e., World Health Organization (WHO) [3], European Network to Advance Best practices & technoLogy on medication adherencE (ENABLE) [10], Ascertaining Barriers for Compliance (ABC) project [11]).

Pharmacists, healthcare providers with expertise in medication use, can be valuable partners in exploring medication non-adherence and providing guidance to patients and other healthcare providers on how to increase adherence levels. Ample research shows significant improvement in medication adherence among diverse types of patients receiving pharmacist-led interventions [12,13,14,15]. Community pharmacists can help provide routine non-adherence screening and assessment, identify non-adherent patients, initiate conversations on reasons for non-adherence, and offer tailored support or services to ensure appropriate and correct use of medications.

Various medication adherence assessment approaches are available to healthcare providers and researchers, including direct and indirect methods. Direct methods, such as medication or metabolite concentration can be costly, invasive, and inconvenient or lead to biased results when patients only take medication before the assessment. Indirect methods, which are more widely used, include pill count, electronic monitoring tools, prescription data (pharmacy dispensing data/physicians prescribing data/insurance claims), and self-reported tools [4,16,17]. Self-reported adherence tools are essential for detecting, understanding, and measuring nonadherence issues and can be highly beneficial in patient care. Numerous tools have been developed, each with its own strengths and weaknesses in terms of completion time, questionnaire complexity, the domains they assess, and their psychometric properties [18,19]. While some tools are more suited for practical application, others, due to their complexity and length, are better suited for research purposes. Some commonly used self-reported tools include the following: The Drug Attitude Inventory questionnaire (DAI), the Morisky Medication Adherence Scale (MMAS-4), the Brief Medication Questionnaire (BMQ), the Hill-Bone Compliance Scale, the Medication Adherence Rating Scale (MARS), the Self-Efficacy Appropriate Medication Use Scale (SEAMS), the Brief Adherence Rating Scale (BARS), the Adherence to Refills and Medications Scale (ARMS), Self Care Inventory Diabetes (SCID), or Screening Tool for AdheRence to medicineS (15-STARS) [16,20,21,22,23,24,25,26,27,28]. There remains a need for a self-assessed adherence questionnaire that is both short and simple enough for practical use while being comprehensive enough to cover several key domains related to adherence and demonstrating good validity. Therefore, the aim of this study is to develop and validate a new self-reported adherence measurement tool that is not only easy and quick to complete but also capable of providing a detailed understanding of patient’s attitudes and behaviors toward medication adherence. The goal is to create a tool that can be simply used in any clinical setting on any type of patient without interfering with the clinician’s workflow, requiring specialty equipment, or causing discomfort to the patient.

## 2. Materials and Methods

### 2.1. Design

**Scheme 1:**
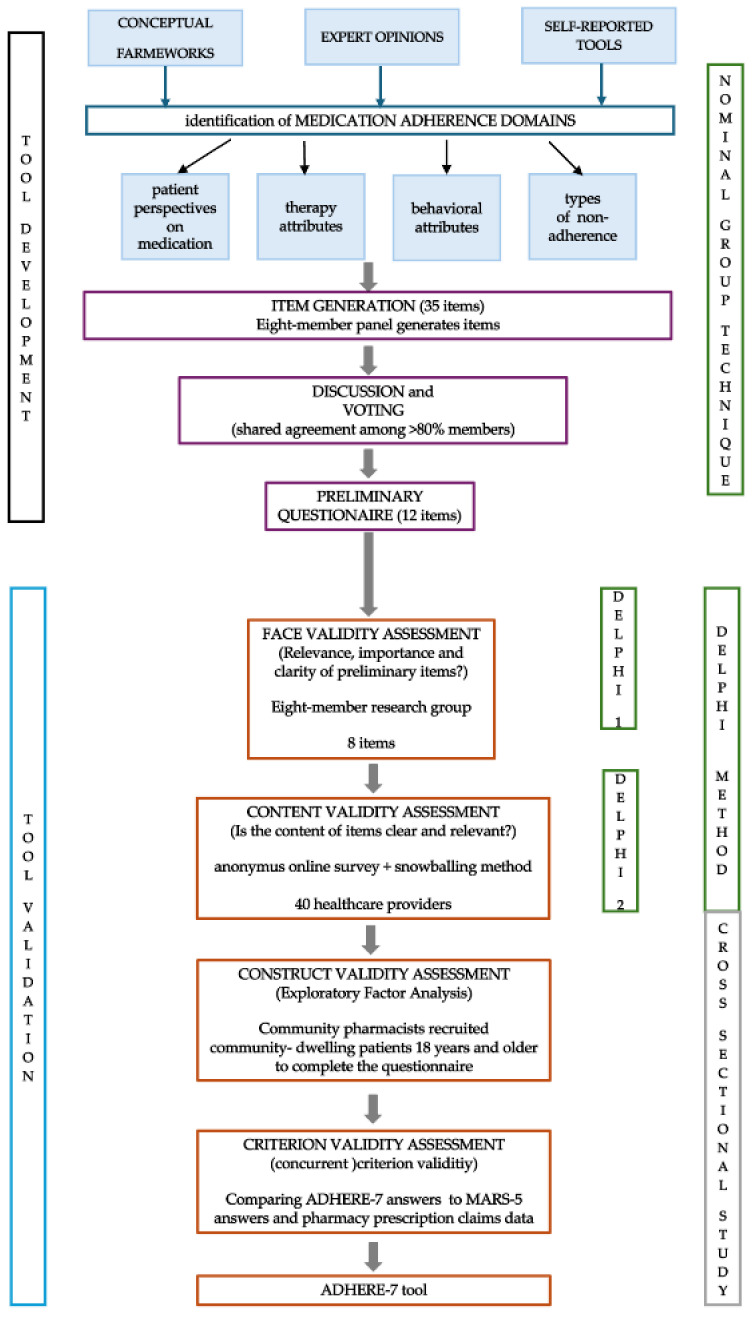
Tool development and validation process.

A two-phase cross-sectional study was conducted from December 2023 to March 2024. The first phase included the development of tool items and involved healthcare professionals. The second phase encompassed a cross-sectional pilot study involving community-dwelling adults in Croatia. Scheme 1 represents the stepwise approach followed during the development and validation process.

### 2.2. Tool Development

Items were generated using a nominal group technique [29]. A ten-member team consisting of experienced academics (three members; MOH, KFŠ, SF), clinical pharmacists (two members; DA, AO), community pharmacists (three members; IB, AGS, AT), a hospital pharmacist (one member; MM), and a physician (one member; MA), participated in item development using inductive and deductive methods [30]. Available medication adherence conceptual frameworks, expert opinions, and studies reporting the use, development, and/or evaluation of self-reported medication adherence tools were studied to collect and identify medication adherence concepts, themes, and determinants [31,32,33,34,35,36,37,38,39,40,41]. Important domains were extracted, such as patient perspectives on medication (harm, utility, appropriateness, availability), therapy attributes (regiment specificities, complexity, cost), behavioral attributes (lifestyle, experiences, health literacy, motivation, cognition), and types of non-adherence (intentional, unintentional). Eight team members were asked to generate up to five questions deemed important and/or used daily in medication adherence assessment in their respective practices. Two team members (MOH, IB) collected the suggestions and compared them to other widely available self-reported tools [25,26,27,28,32,34,42,43,44]. Suggested items were categorized according to the aforementioned domains. A group discussion was held to deliberately generate items with a focus on item linguistic construct and appropriate scoring/answer options. Following the discussion, voting was held, with nine members voting, one member leading and facilitating the discussion (MOH), and one pharmacy student taking notes and counting votes (MB). Item was selected for further inclusion, development, and validation if 80% of members (seven members) agreed on it. Finally, two members of the team (MOH, IB) adapted the items based on voted proposals before forming the preliminary questionnaire (Supplementary Table S1).

### 2.3. Tool Validation

Validation of the Attitudes towards meDication adHErence self-Reported questionnairE (ADHERE-7) included assessment of face validity, content validity, construct validity, criterion validity, internal consistency, and test–retest reliability [45,46,47,48].

#### 2.3.1. Face Validity and Content Validity Assessment through a Modified Delphi Method

A modified Delphi method was used for face and content validity assessment. Firstly, eight members of the research group who participated in item generation assessed the relevance, importance, and clarity of each generated item through a short anonymous online questionnaire using a 5-point Likert scale (strongly agree, agree, neither agree nor disagree, disagree, strongly disagree) formed with Google Forms. Participants could suggest changes for each item. Following the analysis of the first round (face validity), eight items were included in the questionnaire and appropriate phrasing suggestions were incorporated before the subsequent Delphi round (Table S2).

The second Delphi round involved healthcare providers who were not involved in item generation and face validity assessment. Research group members invited registered pharmacists to participate in an anonymous online survey (formed in Google Forms) via the snowballing method. To ensure a high level of expertise and relevant experience, clear selection criteria were established: (1) Minimum practice experience: pharmacists were required to have at least three years of practice experience. (2) Geographic diversity: we sought a diverse geographic representation to capture variations in practice settings. (3) Specialized experience: we prioritized pharmacists with experience in medication adherence counseling or chronic disease management. Team members were instructed to invite peers they believed possessed adequate competence and knowledge on the topic of medication adherence, ensuring they held professional positions that provided suitable insights into the subject matter. The original team members came from diverse settings (i.e., there was no overlap in age, years of professional experience, work location, or educational attainment between team members) which ensured the invitations were sent to participants of miscellaneous sociodemographic backgrounds. Each member invited up to five participants via email, with instructions on how to complete the survey and a request to forward the link to other potential participants. To access the survey, invited participants were presented with a short description of the study’s aim, as well as a digitally authorizable informed consent form, without which they could not access the survey. Participants could access the survey only once ensuring unique inputs were registered. Only complete survey forms were used in the validity assessment. Participants scored each item as clear and relevant using “yes” and “no” answers. The item content validity index (I-CVI) was calculated (agreement on both clarity and relevance was needed to consider the item’s content valid), and items with an I-CVI < 0.80 were revised and reconsidered for exclusion. Additionally, the average content validity index (AVE-CVI) was calculated by summating the I-CVI of all items and dividing by the number of items, and an AVE-CVI > 0.80 indicated the content validity of an entire scale [48]. Considering the commonly applied rule of a participant-to-item ratio of 2:1 to 20:1, a 5:1 ratio was considered for this aspect of validity analysis [49], and a sample of at least 40 professionals was judged adequate, complying with the COnsensus-based Standards for the selection of health Measurement INstruments (COSMIN) checklist [50]. 

#### 2.3.2. Construct Validity 

To identify and bear out the underlying factors, an exploratory factor analysis (EFA) was performed [51]. The choice of rotation method was based on the result of the component correlation matrix and promax rotation was applied. An eigenvalue > 1, a proportion of total variance larger than 60%, and adequate scree plot tests were used as criteria for item/factor retention. The Kaiser–Mayer–Olkin statistic measures were used to determine the sampling adequacy, and Bartlett’s test of sphericity was used to determine the suitability of item/factor reduction. Inter-item and item-scale correlations were explored, and items with low loading values (<0.3) or multiple factor loadings (>0.32) were marked for discussion amongst the research team members, and the final decision on exclusion was made based on consensus. Multiple EFA variations were performed to find the best possible combination of items keeping in mind the complexity of adherence determinants, as well as loading values, variance, and internal consistency. 

#### 2.3.3. Criterion Validity

Concurrent criterion validity was assessed by comparing ADHERE-7 scores with the five-item Medication Adherence Report Scale (MARS-5) scores, with the hypothesis that those identified as having high scores on the MARS-5 scale (higher adherence) would be significantly more likely to show high adherence on the ADHERE-7 scale as well. 

A comparison to pharmacy prescription claims data was used as a second concurrent criterion validity measure. For each participant, pharmacy data on dispensed medication were analyzed and the proportion of days covered (PDC) was assessed. For each patient, the PDC was calculated as the proportion of the sum of days covered in a given period of interest over the number of days in the period of interest of interest. The participant was considered adherent if the calculated PDC ≥ 0.8 [52,53]. The PDC was not calculated for medication used on an as-needed basis, such as analgesics, for medication applied topically, or for medications dispensed for acute conditions. For multiple drug regimens (i.e., three medications taken for hypertension), a day is considered covered when all medications are available to the patient. For each patient, an average PDC was calculated for their entire pharmacotherapy. As pharmacy dispensing software differs among community pharmacies, and different pharmacies collect and store prescription claims in different ways, an interval of at least three months was used to calculate the PDC. The PDC was calculated for a subset of participants, including those who had a minimum of 90 days of prescription claims data available. 

#### 2.3.4. Tool Scoring

All items had a four-answer option. For seven items proposed answers included a negative option and a 3-level scaled positive option (i.e., *yes, 1–2 times a week; yes, 3–4 times a week; yes, 5 or more times; no*). Answering positively (any of the three options) indicated potential non-adherence problems. One item had a 4-point scale (*strongly agree; agree, disagree, strongly disagree*), with disagreement directing towards potential non-adherence. Answers for each item were scored from 1 to 4 with higher scores indicating higher levels of adherence. 

#### 2.3.5. Reliability

Cronbach’s alpha testing was used to determine the internal consistency of the tool. If the exclusion of a certain item led to an increase in internal consistency, that item was removed from the tool. The tool was considered reliable if Cronbach’s alpha was >0.61 [54]. 

#### 2.3.6. Participants

The COSMIN study design checklist for patient-reported outcome measurement instruments was used as a referral point when deciding on the appropriate sample size for each stage of tool validation [50]. A sample of at least 50 participants was chosen for content and criterion validity and internal consistency analysis (6:1 participant-to-item ratio). 

Community pharmacists from geographically distinct regions (central urban Croatia, eastern rural Croatia, southwestern coastal Croatia) were invited to recruit community-dwelling adults (18 years and older) using at least one medication for longer than 2 months to participate in the study. Patients could be offered to participate if they were continuously using the pharmacy’s services for at least three months prior to the study start (checked through the pharmacy’s internal database of prescription claims). Patients were excluded if they were receiving palliative care, had cognitive impairment, or experienced acute worsening of health requiring hospitalization or emergency department visits in the last three days.

### 2.4. Data Analysis

Statistical software IBM SPSS Statistics for Windows Version 26 (IBM Corp., Armonk, NY, USA) was used for data analysis. Sociodemographic data were described using frequencies, percentages, medians, and interquartile ranges (IQR). For all analyses, a value of *p* < 0.05 was considered statistically significant.

## 3. Results

### 3.1. Tool Validation

#### 3.1.1. Face and Content Validity

A total of 42 pharmacists participated in the content validity assessment. The majority of pharmacists were female (85.7%), with a median age of 38.5 years (IQR 34.5–44.25 years), and a median of 13 years of work experience (IQR 8.75–20). Participants mostly worked in community pharmacies (78.6%), and 57.1% of them reported completion of a graduate pharmacy course as the highest educational attainment. Additional information on participants can be found in Table S3. The I-CVI for all items was >0.8, with three items having an I-CVI of 1 (item 3 “I skip or reduce the dose of my medicine, because I am concerned it is causing me harm.”; item 4 “I reduced and/or stopped taking my medicine(s) because it is not helping me.”; and item 6 “How difficult is for you to manage/take care of your medicines?”), while the remaining five items had I-CVIs from 0.95 to 0.976. The AVE-CVI was calculated as 0.975, confirming the content validity of the eight-item scale (Table S4). Participants supported their answers with ten comments, mostly regarding possible answer options (reducing the number of possible answers) or phrasing options they believed would make the item more understandable for patients. 

#### 3.1.2. Construct Validity 

Community pharmacists collected data for 99 patients. Missing data were minimal, with one input having partially incomplete sociodemographic data (1.01% missing data) while all other questions were answered completely. The following characteristics describe the included patients: 64.6% were female, had a median age of 65.5 years (IQR 61–77.25), used a median of five medicines (IQR 3–8) daily, and 56.6% lived in central, urban Croatia. Patients used a variety of medications, mostly medicines for the cardiovascular system (82.83% of patients), alimentary tract, metabolism (65.66% of patients), nervous system (41.41%), and respiratory system (28.28%). Further details can be found in Table 1.

The sample of 99 patients was adequate for exploratory factor analysis, and confirmed with the Kaiser–Meyer–Olkin measure (0.612), while Bartlett’s test of sphericity confirmed factorability (*p* < 0.001). Considering the criteria for item retention and factor formation, seven items were retained forming three factors. Item 2 “I believe I do not need all the medicines prescribed to me.” was removed during EFA due to low loading values and cross-loading (loading values ranging from 0.303 to 0.331). Two items showed cross-loadings, with significantly higher loading values in the original factor, with item 1 (“In the last week, how many times have you missed taking your medicine(s)?”) having loading values of 0.389 and 0.797, and item 6 (“In the past month, how many times have you take your medicine less or less often, due to high costs?”) showing loading values of −0.314 and 0.792. The chosen three-factor extraction analysis explained 73.40% of the variance and left 18% nonredundant residuals with absolute values greater than 0.05. 

The three factors were appropriately labeled: Aversion non-adherence (containing items 4, 5, and 6), Comfort non-adherence (items 1, 3, and 8), and Practical non-adherence (containing item 7). Factors with corresponding items, factor loading values, and item-scale correlations are presented in Table 2. 

#### 3.1.3. Scoring

A simple unweighted approach was chosen. Each of the seven included items could have a score from 1 to 4, and when summed would give the total score for the tool, with higher scores indicating higher levels of reported adherence. 

**Figure 1:**
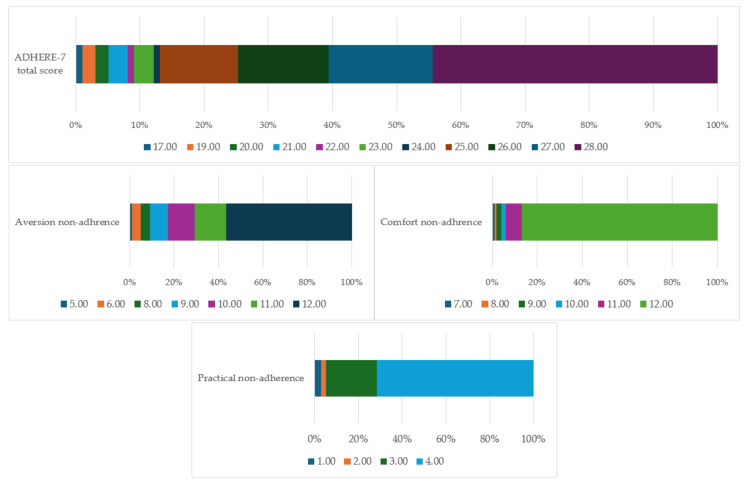
Distribution of factor and tool scores.

The mean total score for the entire ADHERE-7 tool was 26.27 ± 2.41, with a minimum score of 17 and a maximum of 28. For the Aversion non-adherence factor the mean score was 10.89 ± 1.66 (with a range from 5 to 12), for the Comfort non-adherence factor the mean score was 11.74 ± 0.83 (with a range from 5 to 12), and for the Practical non-adherence factor the mean score was 3.63 ± 0.68 (with a range from 1 to 4). Figure 1 depicts the distribution of scores for each factor as well as the tool in total.

#### 3.1.4. Criterion Validity

The ADHERE-7 scores were statistically significantly correlated with the MARS-5 scores, with higher scores on ADHERE-7 being associated with higher scores on the MARS-5 scale (ρ = 0.765; *p* < 0.001). 

For comparison with an objective measurement, the PDC, data were available for 57 of 99 participants. The median values of the ADHERE-7 and PDC were calculated as cut-off points. Values below the median were considered “low adherence”, while values above the median were considered “high adherence”. The gamma rank correlation analysis shows a strong positive correlation between the ADHERE-7 score and PDC values (G = 0.586; *p* = 0.015). The median ADHERE-7 score was 27 (IQR 25–28) and the median PDC value was 0.94 (IQR 0.88–0.99).

#### 3.1.5. Reliability 

Cronbach’s alpha, analyzed to assess internal consistency, showed values of 0.617 and 0.714 for the factors Aversion and Comfort non-adherence, respectively, indicating acceptable and good internal consistency. No item from any factor increased the alpha score when deleted leading to retention of all seven items.

Pharmacists were not instructed to measure the time needed to complete the questionnaire, but the majority reported it took patients less than five minutes for the entire questionnaire and around one minute for the ADHERE-7 tool items. 

### 3.2. Self-Reported Adherence Explored with ADHERE-7 Tool

The most diverse array of answers was given for items 7 and 8, with 23 patients (23.23%) reporting sometimes having problems managing their pharmacotherapy, two reporting issues most days (2.02%), and three having problems every day (3.03%). When asked how many times in the past month they have forgotten to take their medicine, 25 patients (25.25%) reported forgetting once or twice, 8.08% three to four times, and 5.05% reported forgetting to take their medicine(s) five or more times. 

Higher levels of adherence were reported for items 4–6, with a majority of participants answering that they never reduce and/or stop taking their medicine(s) due to perceived harm, lack of effectiveness, or financial cost (92.92%, 90.90%, and 95.95%, respectively).

There was no statistically significant difference in answers between patients with respect to sex, age groups, number of medicines, marital status, or location for any of the items. 

**Figure 2:**
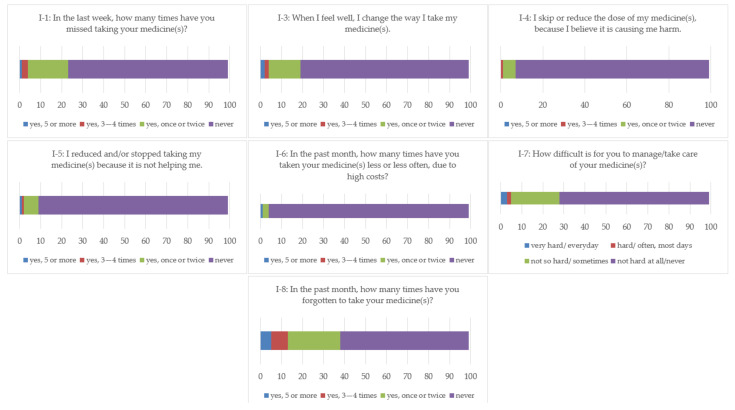
Answers to ADHERE-7.

Figure 2 brings a graphic representation of the answers to the questions within the ADHRE-7 tool.

## 4. Discussion

A new, self-reported medication adherence tool was developed with the aim of helping in the easy and fast assessment of the implementation phase of adherence and potential reasons for non-persistence and discontinuation [55]. Validation analysis indicated acceptable face and content validity, as well as satisfactory criterion validity, with ADHERE-7 scores showing a correlation with both a self-reported tool, such as MARS-5, and an objective measure of PDC collected from the pharmacy prescription claims data. Medication adherence metrics, such as PDC or medication possession ratio, can be useful as a nonadherence screening strategy, and combining results from an objective measure with answers from a short self-reported tool, such as ADHERE-7, can help healthcare providers gain a deeper insight into the patient-important reason for non-adherence [56]. Construct validity analysis revealed items could be grouped into a simple structure of three factors: Aversion, Comfort, and Practical non-adherence. Aversion non-adherence factor examines potential undesirable reasons that led to non-adherence, such as experiencing adverse drug effects, associating medicines with harm or negative experiences, or fear of undesirable consequences. The comfort non-adherence factor explores aspects of adherence associated with oversight or unawareness of non-adherence or the perception that non-adherence is not problematic. Convenient qualities of lifestyle, routine, accessibility, and management self-efficacy are explored with the Practical non-adherence factor. The concept of medication adherence is not only subjective but intricately complex and influenced by numerous interconnected determinants [33]. Healthcare providers view and judge medication adherence with a different objective than patients, leading to potential differences in perception of what constitutes non-adherence as well as the level of importance of reasons for disengaging from any phase of adherence [57,58]. This could explain why items exploring nonintentional and intentional non-adherence (i.e., item 8 and item 3) rotated into the same factor. A qualitative study on patient and healthcare provider perspectives on medication non-adherence supports the complex overlap of different determinants in the model of medication non-adherence [59]. For instance, social determinants of health, such as financial burden or polypharmacy, were viewed as part of unintentional non-adherence, whereas, when using ADHERE-7, financial burden was viewed as part of intentional non-adherence due to disinclination. Regardless, ADHERE-7’s unique factors contribute to a better understanding of non-adherence and are in line with newer theoretical models that explain the behavior of medication adherence [39,60]. 

Items comprising the ADHERE-7 tool cover a range of adherence concepts, including intentional and nonintentional adherence, perception of medication harm or necessity, financial aspects, or capability to manage pharmacotherapy, ensuring an inclusive approach to the topic [61,62]. ADHERE-7 can be positioned or viewed through the Perceptions and Practicalities Approach (PaPA) framework, and addresses both the ability and motivation for adherence, helping healthcare providers and clinicians identify potential triggers and create opportunities to resolve non-adherence [4,63]. It is important to compare and contrast ADHERE-7 to other available tools. For example, SEAMS, ARMS, and MMAS-4 have fewer domains or factors than ADHERE-7, while the Hill–Bone Compliance Scale, DAI, or Personal Evaluation of Transitions in Treatment Questionnaire (PETiT) have substantially more domains, higher complexity, or are medication/disease-specific, limiting their generalizability and simple use [17,21,23,24,26,27,28]. Morisky’s MMAS-4 investigates barriers to adherence but does not delve into reasons for unintentional non-adherence, DAI assesses attitudes but may overestimate compliance, and BMQ can explore self-efficacy but is difficult to score and is intended for patients with comprehensive pharmacotherapy regimens. Tools like SEAMS, 15-STARS, or the OsloMet Adherence to medication Survey (OMAS-37) tool have more items and could require additional time for filing out and final assessment [25,64]. The shortness of the ADHERE-7 tool aids in its practicality for everyday use, increasing the probability that both the clinician and the patient will use it. The tool’s versatility means that different types of healthcare providers and/or researchers can use it. For instance, it can easily be implemented in community pharmacies, primary care physicians’ practices, applied during medication reconciliation in the hospital setting or used by home care nursing staff. ADHERE-7 aims to bridge the gap between practical usability and comprehensive assessment, enhancing the management of medication adherence challenges. Understanding individual patient behavior and attitudes toward medication adherence enables the tailoring of a personalized approach to tackle non-adherence issues. For instance, strategies to tackle “Aversion non-adherence” could include the following: providing detailed patient education about the benefits and risks of medication, recommending and prescribing alternative medications with fewer side effects, or implementing psychological support or counseling to address fears and misconceptions. On the other hand, patients with “Comfort non-adherence” may benefit from using reminders, such as alarms, apps, or pill organizers, education on the importance of consistent medication adherence, and regular follow-ups and monitoring to reinforce adherence behaviors. Moreover, strategies to tackle “Practical non-adherence” could include simplifying medication regimens (e.g., once-daily dosing) and ensuring medications are affordable and accessible. 

### Strengths and Limitations

The development and validation of the ADHERE-7 tool were performed on a sample of community-dwelling patients from one country and in one language which can be characterized as a limitation, inhibiting potential generalization to other/different cultural health groups. Cultural, linguistic, and social factors can influence participants’ patterns of medication use and consequently participants’ understanding of non-adherence [65]. Participants’ characteristics, including a wide range of ages (19–95 years), educational attainment, employment status, and number of used medicines (1–16 medicines), as well as fitting reliability analysis and adequate Kaiser–Meyer–Olkin measure, indicate that the sample was suitable for analysis. While the size of the test group involved (40 for pharmacists and 99 for patients) could be judged as relatively small, the COSMIN study design checklist for patient-reported outcome measurement instruments deliberates these sample sizes as adequate and very good for the purposes of content, structural, and construct validity assessment, which additionally confirms sample size suitability. A further limitation could concern the type of participating patients and the potential lack of diversity in answers to the questionnaire. Participants were patients with common chronic conditions, such as cardiovascular diseases, treated within a primary care setting and reporting high adherence. Patients with predominantly different comorbidities may have different adherence-related barriers [62] and this tool should be used in various settings to further confirm its advantage as a non-disease-specific tool. All items in the ADHERE-7 tool are non-disease- and non-medication-specific, ensuring the tool can be appropriate for use in all patients, settings, and medications. Straightforward wording warrants easy cultural and linguistic adaptations. Future applications of the tool could include the use of ADHERE-7 in both research and healthcare settings, as well as possible integration of the questionnaire as a part of newer digital technologies (i.e., telemedicine, mobile applications, medication management tools…) to help monitor and improve medication adherence [10,66].

When comparing ADHERE-7 to other available tools that explore medication adherence in a more comprehensive manner, a merit in the form of shortness needs to be stated [64], without compromising the ability to explore various important non-adherence contexts. 

The majority of participants reported high medication adherence, which could be viewed as sampling or nonresponse bias given the data were collected in community pharmacies and non-adherent patients would be less likely to visit the pharmacy on a regular basis. Nonetheless, even amongst included participants, the ADHERE-7 tool aided in the identification of those with suboptimal adherence. ADHERE-7 scores correlated both with a subjective self-reported measure (MARS-5) and an objective measure (PDC), indicating satisfactory sensitivity and specificity.

## 5. Conclusions

This study involved the development of a new self-reported medication adherence tool, ADHERE-7, which demonstrated satisfactory psychometric performance. ADHERE-7 is concise and user-friendly while offering comprehensive insights into patient behavior and prospective reasons regarding medication non-adherence. It has the potential to be used as a part of routine medication adherence screening in diverse clinical settings, allowing pharmacists, physicians, and other healthcare providers to examine or confirm potential non-adherence. ADHERE-7 addresses various aspects, such as aversion, comfort, and practical issues, as well as intentional and unintentional nonadherence. This tool can enhance our understanding and management of medication nonadherence, a well-researched but unresolved issue.

## Figures and Tables

**Table 1:** Participants’ characteristics.

Characteristics	n = 99
Sex (female, %)	64.6%
Age (median, IQR)	65.5 (61–77.25)
Medicines (median, IQR)	5 (3–8)
Educational attainment (%)	
primary education ^a^ secondary education ^b^ community college ^c^ university degree	21.4% 51.0% 12.2% 15.3%
Employment status (%)	
employed unemployed retired	34.7% 1% 64.3%
Marital status (%)	
married common-law marriage divorced widowed single	52% 7.1% 4.1% 29.6% 7.1%
Location (%)	
central urban eastern rural southwestern costal	56.6% 28.3% 15.2%
Prescribed medicines based on ATC classification system (%)	
cardiovascular system alimentary tract and metabolism nervous system respiratory system antiinfectives for systemic use blood and blood forming organs genitourinary system and sex hormones musculo-skeletal system sensory organs systemic hormonal preparations dermatologics antineoplastic and immunomodulating agents	82.83% 65.66% 41.41% 28.28% 18.18% 14.14% 14.14% 12.12% 12.12% 11.11% 9.09% 7.07%

^a^ mandatory eight-year education ^b^ four-year education ^c^ two- or three-year higher education course, ATC—Anatomical Therapeutic Chemical, IQR—interquartile ranges.

**Table 2:** Factors and corresponding items.

Factor	Item Code	Item	Factor Loading	Item-Scale Correlation
Aversion non-adherence (Cronbach’s α 0.617)	I-4	I skip or reduce the dose of my medicine(s), because I believe it is causing me harm.	0.745	0.569
	I-5	I reduced and/or stopped taking my medicine(s) because it is not helping me.	0.725	0.452
	I-6	In the past month, how many times have you taken your medicine(s) less or less often, due to high costs?	0.792	0.307
Comfort non-adherence (Cronbach’s α 0.741)	I-1	In the last week, how many times have you missed taking your medicine(s)?	0.909	0.613
	I-3	When I feel well, I change the way I take my medicine(s).	0.797	0.616
	I-8	In the past month, how many times have you forgotten to take your medicine(s)?	0.726	0.540
Practical non-adherence	I-7	How difficult is for you to manage/take care of your medicine(s)? (Managing/taking care of medicines includes all actions related to ordering, picking up, preparing and taking medicine(s))	0.881	n/a

## Data Availability

No new data were created or analyzed in this study. Data sharing is not applicable to this article.

## Supplementary Materials

The following supporting information can be downloaded at: https://www.mdpi.com/article/10.3390/pharmacy12040113/s1; Table S1: Preliminary questions for ADHERE tool development and validation; Table S2: Preliminary 8 questions for the ADHERE tool; Table S3: Characteristics of participants involved in content validity assessment; Table S4: Item content validity index and scale content validity.
