# Supplementary material for: Development and Validation of a Tool to Explore Attitudes Towards meDication adHErence Using a Novel Self-Reported QuestionnairE (ADHERE-7)
Source: Pharmacy (Basel). 2024 Jul 18;12(4):113. doi: 10.3390/pharmacy12040113 (PMC11270287; doi:10.3390/pharmacy12040113)
Supplement: Supplementary file 1 [file pharmacy-12-00113-s001.zip › pharmacy-3064623-supplementary.pdf]

Table S1: Preliminary questions for ADHERE tool development and validation

item suggestion	answer	themes/domains	further development	predeveloped version
Do you think you are prescribed too many medicine(s)	yes/no	therapy attributes ,patient perspectives on medication, therapy attributes	yes	**In the last week, how many times have you missed taking your medicines?**
Do you use reminders to help you take your medicine(s)?	yes/no	therapy attributes	no	
What do you do when you forget to take your medicine(s)?	open ended	behavioral attributes, types of non-adherence	no	
How do you manage your therapy given the number of medicine(s) you need to take?	open ended	patient perspectives on medication, behavioral attributes, therapy attributes, types of non-adherence	no	
Do you think a medicine(s) dispenser or medicine(s) list would be useful?	yes/no	therapy attributes, behavioral attributes	no	
Do you understand the purpose and method of using your medicine(s)?	yes/no	patient perspectives on medication, behavioral attributes	no	**I believe I do not need all the medicines prescribed to me.**<br>**I do not need to take all of my medicines.**
Do you have difficulties in taking your medicine(s)?	yes/no	behavioral attributes, therapy attributes, patient perspectives on medication	yes	
Have you forgotten to take your therapy in the last month?	yes/no	types of non-adherence	yes	
In the last month, have you skipped your dose due to high cost of your medicine(s) to make the medicine(s) "last longer"?	yes/no	therapy attributes, behavioral attributes, types of non-adherence, patient perspectives on medication	yes	
Do you believe that therapy helps you?	yes/no	therapy attributes ,patient perspectives on medication	yes	**When I feel well, I omit, skip or reduce the dose of my medicine(s), even though it/that was not recommended.**
Are you using these medicine(s) for the first time?	yes/no	patient perspectives on medication, behavioral attributes	no	
This medicine(s) is prescribed to be taken as needed. How do you use it? How often is "as needed" for you?	open ended	behavioral attributes, types of non-adherence	no	
Do you understand what the medicine(s) is for?	yes/no	behavioral attributes, therapy attributes	no	
Do you know how long you should take these medicine(s)?	yes/no	patient perspectives on medication, therapy attributes	no	
Have you noticed that any of these medicine(s) are not working for you?	yes/no	therapy attributes, patient perspectives on medication, types of non-adherence, behavioral attributes	yes	**I skip, reduce, or stop taking my medicine, despite the recommendation, because I am concerned that the medicine is causing me harm**
Do you know why these medicine(s) have been prescribed for you?	yes/no	therapy attributes	no	
How often do you skip or not take a medicine(s) that you use regularly	time scale	types of non-adherence	yes	
Do you have any reason why you skip taking the medicine(s)? / Why do you miss taking your medicine(s)?	open ended	behavioral attributes, patient perspectives on medication	yes	
Do you change the way you take the medicine(s) compared to what was prescribed by your doctor? For example, do you only take half a tablet instead of the whole one?	yes/no	patient perspectives on medication, behavioral attributes	yes	**I reduced and/or stopped taking my medicine(s) because it is not helping me.**<br>**I reduced and/or stopped taking my medicine because it is not effective.**
Do you use the medicine(s) regularly according to the instructions provided?	yes/no	therapy attributes, therapy attributes, types of non-adherence	yes	
Do you forget to use your medicine(s)?	yes/no	types of non-adherence	yes	
Do you stop taking medicine(s)s in situations where you think you don't need them?	yes/no	patient perspectives on medication, behavioral attributes	yes	**Taking medicine(s) regularly is a big expense for me, so I take them less or less often than recommended.**<br>**Taking my medication as prescribed is a significant expense, so I take them less or less often than I should.**
Do you stop using the medicine(s) when you don't feel well?	yes/no	patient perspectives on medication, behavioral attributes, types of non-adherence	yes	

Taking medicine(s) creates additional costs for you, and as a result you take the medicine(s)/medicine(s) less often than recommended	yes/no	patient perspectives on medication, therapy attributes	yes	**I forget to take my medicine(s) because I am to busy with other things in my life**
Do you forget to take the medicine(s) if you are on the road/ when you are not at home	yes/no or time scale	therapy attributes, behavioral attributes, types of non-adherence	yes	
You forget/find it difficult to remember to take the medicine(s) every day	yes/no	therapy attributes, behavioral attributes, types of non-adherence	yes	
You think you are taking too many medicine(s)s	Likert scale	patient perspectives on medication, therapy attributes	yes	**Do you forget to take your medicine(s)?**
How sure are you that the medicine(s) is doing you good?	Likert scale	patient perspectives on medication	yes	
How sure are you that the medicine(s) harms you?	Likert scale	patient perspectives on medication	yes	
You believe that the medicine(s) does not help you/is not effective	Likert scale	patient perspectives on medication	yes	
how many times have you missed taking a dose of your medicine(s) in the last week?	time scale	types of non-adherence	yes	
How do medicine(s) fit into your everyday life schedule?	open ended	patient perspectives on medication, behavioral attributes	yes	**I find it difficult to manage my medicine(s) so I use them less often or less than recommended**
Do you understand why you were prescribed these medicine(s)	yes/no	patient perspectives on medication, behavioral attributes	no	
Do you ever intentionally skip taking your medicine(s)?	yes/no	types of non-adherence	yes	**I do not take my medicine(s) as prescribed because it is difficult for me to remember every instruction.**
Do you have problems managing your medicine(s)?	yes/no	patient perspectives on medication, therapy attributes, behavioral attributes	yes	

Table S2 Preliminary 8 questions for the ADHERE tool

Item code	Croatian version	English version
I-1	Koliko ste puta propustili uzeti terapiju u posljednjih tjedan dana?	*In the last week, how many times have you missed taking your medicines?*
I-2	Smatram da mi nisu potrebni svi propisani lijekovi.	*I believe I do not need all the medicines prescribed to me.*
I-3	Kad se osjećam dobro izostavim ili smanjim uzimanje propisanog lijeka	*When I feel well I change the way I take my medicine.*
I-4	Preskočim ili smanjim dozu lijeka, jer smatram da mi lijek šteti.	*I skip or reduce the dose of my medicine, because I believe it is causing me harm.*
I-5	Smanjio/la ili prekinuo/la sam uzimanje nekog lijeka, jer smatram da mi ne pomaže.	*I reduced and/or stopped taking my medicine(s) because it is not helping me.*
I-6	Koliko ste puta u posljednjih mjesec dana zbog velikih troškova, lijek koristili rjeđe ili manje?	*In the past month, how many times have you take your medicine less or less often, due to high costs?*
I-7	Koliko Vam je teško voditi brigu o Vašoj terapiji? (Briga o terapiji uključuje sve radnje povezane s naručivanjem i preuzimanjem lijekova, svakodnevnim pripremanjem i primjenom lijeka)	*How difficult is for you to manage/take care of your medicines? (Managing/taking care of medicines includes all actions related to ordering, picking up, preparing and taking medicine(s))*
I-8	Koliko ste puta u posljednjih mjesec dana zaboravili uzeti svoju terapiju?	*In the past month home many times have you forgotten to take your medicine(s)?*

Table S3 Characteristics of participants involved in content validity assessment

**Participants' characteristic**	**n=42**
sex (female, %)	36; 85.7%
age (median, IQR)	38.5 (34.5-44.25)
years of experience (median, IQR)	13 (8.75-20)
highest educational attainment	
graduate degree<br>postgraduate specialist course[a]<br>health specialization[b]<br>doctoral degree (PhD)	24; 57.1%<br>12; 28.6%<br>4; 9.5%<br>2; 4.8%
practice placement	
community pharmacy<br>hospital pharmacy<br>industry	33; 78.6%<br>6; 14.3%<br>3; 7.1%
[a] 1-year course, [b] 3-year healthcare residency including postgraduate specialist course, IQR—interquartile range	

Table S4 Item content validity index and scale content validity

Item	item clarity	item relevance	item- content validity index
I-1: In the last week, how many times have you missed taking your medicines?	41	42	0.975
I-2: I believe I do not need all the medicines prescribed to me.	40	42	0.95
I-3: When I feel well I change the way I take my medicine.	41	42	0.976
I-4: I skip or reduce the dose of my medicine, because I believe it is causing me harm.	42	42	1
I-5: I reduced and/or stopped taking my medicine(s) because it is not helping me.	42	42	1
I-6: In the past month, how many times have you take your medicine less or less often, due to high costs?	40	41	0.95
I-7: How difficult is for you to manage/take care of your medicines? (Managing/taking care of medicines includes all actions related to ordering, picking up, preparing and taking medicine(s))	42	42	1
I-8: In the past month home many times have you forgotten to take your medicine(s)?	41	40	0.95
average content validity index (scale validity)	0.975		
